# Neurophysiological study to assess the severity of each site through the motor neuron fiber in entrapment neuropathy

**DOI:** 10.1186/1749-7221-4-7

**Published:** 2009-06-17

**Authors:** Ryoichi Shibuya, Hideo Kawai, Kouji Yamamoto

**Affiliations:** 1 Department of Rehabilitation, Osaka Rosai Hospital, Sakai, Japan; 2 Department of Orthopaedic Surgery, Hosigaoka Kouseinenkin Hospital, Hirakata, Japan; 3 Department of Orthopaedic Surgery, Toyonaka Municipal Hospital, Toyonaka, Japan

## Abstract

**Background:**

The double crush hypothesis (DCH) that had been widely accepted seems to have been dismissed recently. Prior to the DCH, retrograde changes in the proximal median nerve in carpal tunnel syndrome (CTS) were reported. There has been no report of quantitative analyzing about the effect of one site's compression on another site all through the same peripheral nerve in CTS patients.

**Methods:**

We measured the central motor conduction time (CMCT), motor conduction latency of the cervical root region (CRL), peripheral path latency from the rootlet to the wrist (PL) and motor distal latency (MDL) in the median nerve and ulnar nerves, respectively in CTS patients.

**Results:**

MDL, PL and CRL were prolonged selectively in the median nerve, but not in the ulnar nerve of CTS patients. And in the median nerve measurement, MDL was high (r = 0.59, p < 0.0001) while PL showed a significant (r = -0.28, p < 0.05) relationship with CRL. MDL was large (r = 0.58, p < 0.0001) and showed a close (r = 0.59, p < 0.0001) relationship with the amplitude of CMAP. There was no significant difference between the amplitude of the normal CRL group and that of the prolonged CRL group. This quantitative analysis showed a linear relationship among MDL, CRL and CMAP amplitude.

**Conclusion:**

Dual entrapment lesions did not unexpectedly exaggerate the vulnerability or total damage. The vulnerability and the damage were proportional to the severity of each lesion. If the DCH term presented to an unexpectedly exaggerated degree, the cases of double crush symdrome in the CTS patients were rare, but if the term DCH refers to only this linear relationship, the DCH should not be dismissed.

## Background

In 1973, Upton and McComas found that cervical radiculopathy coexisted in 81 of 115 patients with carpal tunnel syndrome (CTS) and/or ulnar neuropathy at the elbow (UNE) [1]. They proposed a hypothesis that more than one subclinical focal compression lesion on the same nerve fiber could cause susceptibility at a distal commpression site and exaggerate the damage. This double crush hypothesis (DCH) has been accepted in many experimental and clinical reports [2-7]].

These previous reports attempted to verify two pathological factors as follows: #1: A focal compression on the nerve fiber influences on the vulnerability of the other regions along the same fiber [2,3,6-8]. #2: The dual regions of compression on the nerve fiber exaggerate the total damage expected [4,5,9-11].

Wilbourn and Gilliatt pointed that #1 nor #2 could not be validated in previous experimental reports [12] and Morgan and Wilbourn reported that the double crush syndrome (DCS) was rare in carpal tunnel syndrome (CTS) patients for anatomical and pathological reasons [13]. Kwon et al. revealed that C6, C7 radiculopathy had no significant influence on sensory responses while C8 radiculopathy had no significant influence on the motor disturbance in CTS patients; they did not support the DCH[14]. Recently DCH seems to have been dismissed.

Retrograde changes on the proximal fibers of median nerves were found in CTS patients [15-18]. We doubted whether the influence of another nerve-fiber site could be neglected in the clinical diagnosing of the paresis.

In the current study, we non-invasively measured central motor conduction time (CMCT), motor conduction latency of the cervical root region (CRL), peripheral latency between the rootlet and the wrist (PL), motor distal latency (MDL) through the transverse ligament (MDL) and amplitude of compound muscle action potential (CMAP) in CTS patients.

We quantitatively evaluated disturbances all along the peripheral nerve with compression lesions and examined the DCH.

## Subjects

CTS was diagnosed based on the presence of symptoms such as numbness, tingling, clumsiness, or nocturnal symptoms of burning/cold, tightness, sore/ache/discomfort, or puffiness with exacerbation in median nerve distribution. The diagnosis was often supported by a positive Phalen's or Tinel's sign, thener muscle weakness or atrophy, but these signs were not required. One hundred seventy-four hands of 114 patients were diagnosed as having idiopathic CTS. Sixty patients were bilaterally affected. Fifty four patients had CTS hands on the unilateral side and 54 contra-lateral hands were asymptomatic. The diagnosis of CTS in all patients was reconfirmed at our clinic, but in 83 of 114 patients, the plain roentogenograms of cervical spine were taken before they came to our clinic. No patient had a history of epilepsy, heart disease treated with a pacemaker or stent, intracranial anurysm clips or metel.

## Methods

(1) Patients were seated with their upper arms relaxed.

Cathode electrodes were placed on the motor points of the abductor pollicis brevis (APB) and the abductor digiti minimi (ADM) muscles. The reference electrodes were placed on the tendons of the APB and ADM.

Following a per cutaneous electric stimulation to the median nerve and the ulnar nerve at the wrist 7 cm proximal to the cathode electrode, M-waves and F-waves were recorded with a bandwidth of between 5 Hz and 10 kHz using a Neuropack Four (MEM-4104, Nihon Kohden, Japan). At least 20 per cutaneous electric stimulations were delivered at the same sites and the M-waves and F-waves of the earliest onset latency were recoded for the calculations. The amplitude of compound muscle action potential (CMAP) was obtained by measuring the peak to peak of the M-wave. Motor distal latency (MDL) was obtained from the onset latency of the M-wave. The latency between the anterior cells and the muscle was calculated according to the formula of Kimura [19] as follows:

(2) Motor evoked potential (MEP) following trans-cranial (MEPcr) and cervical (MEPcv) electromagnetic stimulation was recorded via the same electrodes on APB and ADM by magnetic stimulation (STM-1200, Nihon Kohden, Japan). We used a 15 cm (inner diameter) round coil (YM-101) for the trans-cranial stimulation as described in detail by Barker et al [20]. In order to stimulate the motor roots we used a 7 cm (inner diameter) round coil (YM-102). The central motor conduction time (CMCT) was calculated by subtracting the latency between the anterior cells and the muscle from the onset latency of MEPcr.

The latency from the anterior horn cells to the rootlet (CRL) was calculated by subtracting the onset latency of the MEPcv from the latency between the anterior cells and the muscle. Peripheral latency (PL) defined the latency from rootlet to wrist was obtained by subtracting CRL and MDL from the latency between the anterior cells and the muscle. All procedures were performed with informed consent from patients and with approval of our institute ethics committee.

Plain roentogenograms of the cervical spine in 83 patients were reviewed. In the rentogenogram review, spondylotic change such as narrowing of intervertebral discs, osteophytosis around disc margins and facet joints were evaluated by the author, who had no knowledge of the symptoms or electro-physiological measurements.

### Statistics

All values for each group are presented as means + standard deviation (SD) using statistical software (Statview 4.5J, SAS, Cary, NC). The significance of differences between the values was analyzed using the Mann-Whitney U-test (Stadview 4.5J) and a simple regression model (Stadview 4.5J) was used to evaluate the correlation between the measured data. All P values less than 0.05 were considered to indicate statistical significance.

## Results

Measurement of 54 hands on the asymptomatic side

CMCT, CRL, PL and MDL were measurable in all 54 hands on the asymptomatic side.

In median nerve measurement, CMCT was 7.05 ± 0.99 ms, CRL was 1.10 ± 0.38 ms, PL was 8.67 ± 0.84 ms and MDL was 3.80 ± 0.51 ms. The amplitude of the M- wave was 11.4 ± 3.18 mV.

Because the mean ± SD of CRL was 1.10 ± 0.38 ms in this study, the normal range of CRL was defined as a value ranging from 0.72 ms to 1.48 ms. And the CRL that was longer than 1.48 ms was defined to be prolonged CRL. The normal range of CMAP amplitude was defined a value ranging from 8.2 mV to 14.6 mV. These normal values were used in an analysis of the symptoms in the following sections.

In ulnar nerve measurement, CMCT was 7.11 ± 0.98 ms, CRL was 1.19 ± 0.40 ms, PL was 8.48 ± 0.89 ms and MDL was 2.48 ± 0.21 ms. The amplitude of the M- wave was 7.77 ± 2.32 mV.

No relationship was recognized among the CMCT, PL, CRL and MDL of the median nerve or ulnar nerve. MDL, PL and CRL showed no significant relationship with the CMAP amplitude or the APB muscle. In measurements of 54 asymptomatic hands, there was no significant difference between the median nerve and ulnar nerve.

Measurement of 174 hands on the symptomatic side

In symptomatic174 hands, the F wave could not be elicited in19 hands and the M wave could not be elicited in 12 hands following median nerve stimulation.

The other143 hands had complete data for all CMCT, CRL, PL and MDL.

In median nerve measurement, CMCT was 7.15 ± 1.37 ms, CRL was 1.72 ± 0.73 ms, PL was 9.25 ± 1.06 ms and MDL was 6.26 ± 1.75 ms. The amplitude of the M- wave was 6.35 ± 3.91 mV.

In ulnar nerve measurement, CMCT was 6.98 ± 1.41 m, CRL was 1.22 ± 0.41 ms, PL was 8.75 ± 0.97 ms and MDL was 2.53 ± 0.25 ms. The amplitude of the M- wave was 7.56 ± 2.14 mV.

In 143 symptomatic hands, MDL, PL and CRL of the median nerve were significantly (p < 0.0001) longer than those of the ulnar nerve. CMCT of the median nerve did not show a significant difference from that of the ulnar nerve.

**Figure 1:**
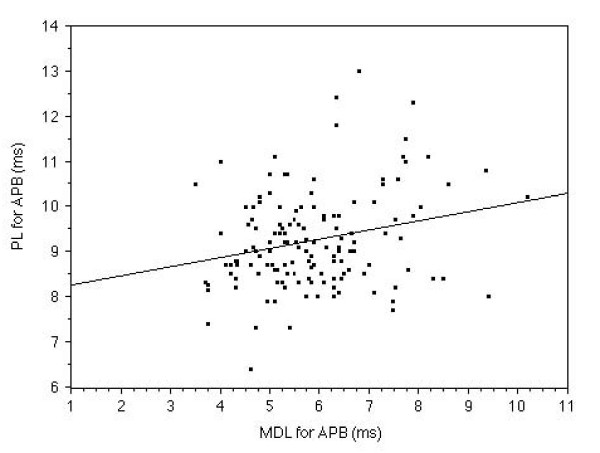
**The relationship between MDL and PL in the median nerve measurement**. Relationship between MDL and PL measured in the median nerve of 143 symptomatic hands is shown. The MDL demonstrated a significant (r = 0.28, p < 0.005) relationship with the PL.

**Figure 2:**
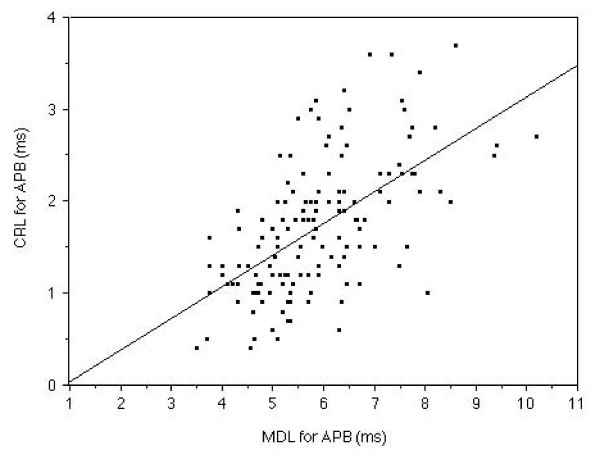
**The relationship between MDL and CRL in the median nerve measurement**. Relationship between MDL and CRL measured in the median nerve of 143 symptomatic hands is shown. The symptomatic MDL demonstrated a strong (r = 0.28, p < 0.005) relationship with the CRL.

MDL of the median nerve showed a significant relationship (r = 0.28, p < 0.005) with PL of the median nerve. MDL of the median nerve demonstrated a strong relationship (r = 0.59, p < 0.0001) with CRL of the median nerve. (Fig. 1 and Fig. 2)

MDL of the ulnar nerve demonstrated a significant relationship (r = 0.31, p < 0.001) with PL of the ulnar nerve, but did not demonstrate a significant relationship with CRL. CMCT showed no relationship with MDL in median or ulnar nerve measurements.

**Figure 3:**
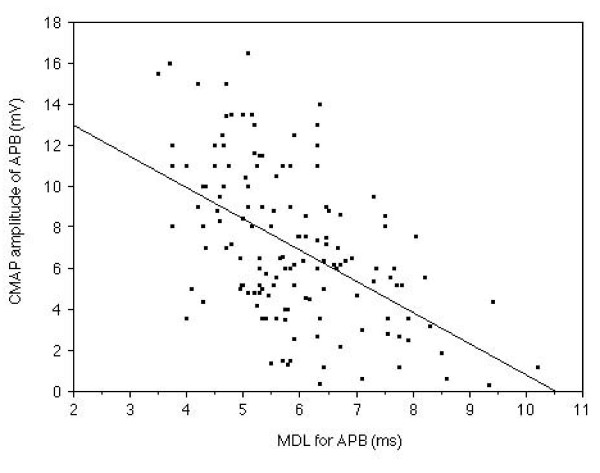
**The relationship between MDL and CMAP amplitude of the APB muscle**. Relationship between MDL and CMAP amplitude measured in the median nerve of 143 symptomatic hands is shown. The MDL demonstrated a strong (r = 0.58, p < 0.0001) relationship with the CMAP amplitude.

**Figure 4:**
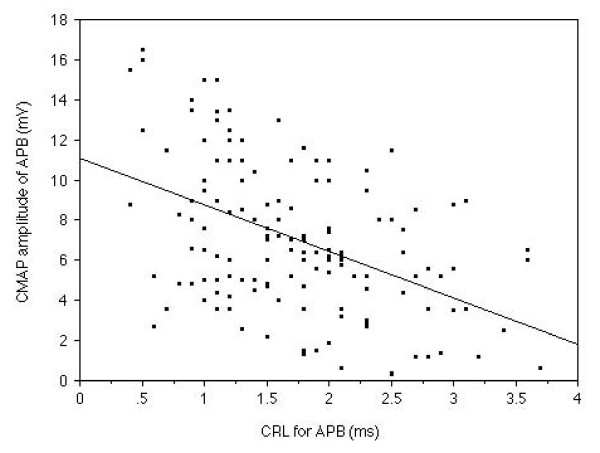
**The relationship between CRL and CMAP amplitude of the APB muscle**. Relationship between CRL and CMAP amplitude measured in the median nerve of 143 symptomatic hands is shown. The CRL demonstrated a strong (r = 0.58, p < 0.0001) relationship with the CMAP amplitude.

In the median nerve of symptomatic hands, MDL demonstrated a strong (r = 0.58, p < 0.0001) relationship with the amplitude of CMAP following median nerve stimulation at the wrist. (Fig. 3) CRL of the median nerve showed a close (r = 0.59, p < 0.0001) relationship with the amplitude of CMAP. (Fig. 4)

We classified the 143 measurable symptomatic hands into two groups according to the value of the CRL measured in the median nerve. One was a normal CRL group (n = 59 hands) and the other was a prolonged CRL group (n = 84 hands).

The MDL of the normal CRL group was 5.12 ± 0.83 ms and that of the prolonged CRL group was 6.38 ± 1.48 ms.

**Figure 5:**
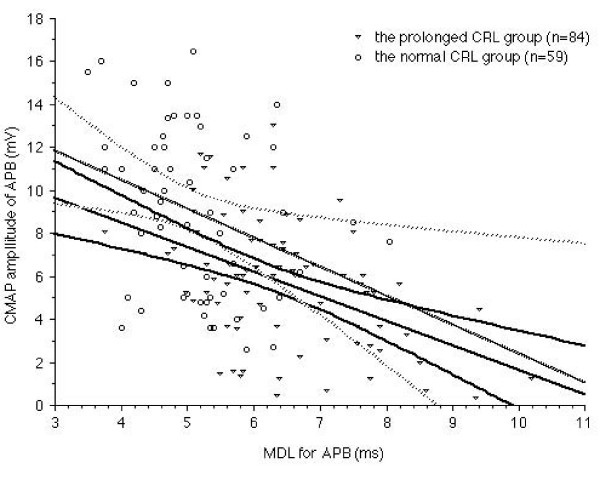
**The relationship between MDL and CMAP amplitude of APB muscle in the prolonged CRL group and normal CRL group**. Relationship between MDL and CMAP amplitude is shown. Normal CRL group (open circles) and prolonged CRL group (open triangles). The dotted line represents regression and 95% confidence intervals between MDL and amplitude of CMAP in the normal CRL group (n = 59, r = -0.32, p < 0.05). The solid line demonstrates regression and 95% confidence intervals between MDL and amplitude of CMAP in the prolonged CRL group (n = 84, r = -0.46, p < 0.0001). There was no significant difference between the two groups.

In the normal CRL group, MDL showed a significant (r = -0.32, p < 0.05) relationship with the amplitude of CMAP. In the prolonged CRL group, MDL showed a good (r = -0.46, p < 0.0001) relationship with the amplitude of CMAP. There was no significant difference between the amplitudes of CMAP in the normal CRL group and those of the prolonged CRL group.(Fig. 5)

The plain roentogenograms demonstrated spondyolotic changes in 60 of 83 patients. There was no significant difference between values of CMCT, CRL, PL or MDL for 120 hands of patients with spondylosis and those of 46 hands without spondylotic changes in either median or ulnar nerve measurements.

## Discussion

The typical symptoms of cervical myelopathy are numbness and clumsy hands [21-23] In addition, Friedenberg and Miller found that degenerative changes at the cervical spine appear in 75% of asymptomatic patients by the seventh decade [24]. Teresi et al. took MR images in asymptomatic patients and found spinal cord impingement in 16% of patients under 64 years of age and in 26% of those over 64 years [25]. Bednarik et al. measured motor evoked potential by transcranial and root magnetic stimulation and reported that the sensitivity of MEP was 40% for subclinical cervical compression myelopathy [26]. Lo et al. demonstrated an excellent correlation between Transcranial magnetic stimulation (TMS) findings and MRI image and said in their article "TMS can be recommended as a non-invasive, less costly, and less time-consuming technique for screening and serial evaluation of cervical spondylotic myelopathy [27]. We consider it preferable to detect disorders of the cervical spine in the diagnosis of the CTS.

In the current study, we measured CMCT which had no relationship with the severity of peripheral nerve lesions, because we thought the CMCT represents the function of the central motor fibers which are not continuous at the anterior-horn.

Upton and McComas found that under a condition in which more than one focal nerve compression exists along one nerve fiber, this fiber increases its susceptibility at a distal site and the degree of damage at the distal compression site on the same nerve fiber. They attributed this condition to a disturbance in axonoplasmic flow at the proximal compression site, and proposed a double crush hypothesis (DCH). This DCH was accepted in many experimental and clinical reports [2-7].

Wilbourn et al. pointed out that the DCS of cervical radiculopathy and CTS is rare because 1) Preganglionic sensory fibers and postganglionic sensory fibers are not anatomically continuous at the dorsal root ganglion. 2) The sensory fiber of the median nerve that transverses the carpal tunnel originates from three separate cervical roots (C6, C7, C8) and its motor fiber of that from C8 and Th1 roots. 3) Damage due to the axonoplasmic flow disturbance that Uptom and McComas advocated is axonal loss, while the compression neuropathy is pathologically demyelination [12].

Kwon et al. compared the CTS patients with C6 or C7 radiculopathy and those with C8 radiculopathy in terms of the motor and the sensory latencies and amplitudes of the median nerve and found no significant differences between them. Therefore cervical radiculopathies have no significant influence on the severity of CTS. Kwon et al. concluded that the DCS hardly existed in idiopathic CTS [14].

Prior to the DCH, Thomas and Fullerton described a change in the peripheral nerve proximal to the wrist in CTS patients [15]. Pease et al showed a reduction in the median nerve conduction velocity at the forearm in CTS patients [16]. Anastasopoulos and Uchida demonstrated a delay in the F-wave of the median nerve in CTS [17,18]. Stoehr and colleagues found that the extent of the retrograde changes correlated with the degree of CTS severity [28].

The sensory fibers of the median nerve that transverses the carpal tunnel originates from three separate cervical roots (C6, C7, C8), while the sensory nerve of the ulnar nerve originates from two separate cervical roots (C8, Th1). The motor fiber of the median nerve originates from two separate cervical roots (C8, Th1) in much the same way as the ulnar nerve, so in order to analyse the DCH, we considered the motor fiber and measured the CMCT, CRL, PL and MDL between the median nerve and ulnar nerve in each case. No report has quantitatively evaluated the correlation between CRL, PL and MDL.

As Wilbourn et al. mentioned, we thought that the DCH requires two pathologies to be verified and many previous reports attempted to prove these two pathologies as follows: #1: A focal compression on the nerve fiber influences the vulnerability of other regions along the same fiber [2,3,6-8].

#2: Dual regions of compression on the nerve fiber exaggerate the total damage making it more severe than expected [4,5,9,11]. We tested #1 and #2 in CTS patients.

### Discussion about #1

A focal compression on the nerve fiber influences the vulnerability of the other regions along the same fiber.

In 143 of 173 measurable symptomatic hands, MDL, PL and CRL of the median nerve were a significantly longer than those of the ulnar nerve. MDL demonstrated a significant relationship with PL (r = 0.28, p < 0.005) and a strong relationship with CRL (r = 0.59, p < 0.0001) in this study (Fig. 1 and Fig. 2) in the median nerve measurement. However in the ulnar nerve, MDL showed a significant relationship with only PL (r = 0.31, p < 0.001). The MDL of the ulnar nerve did not demonstrate significant relationship with CRL. These facts suggest that the median nerve was selectively damaged all along the motor fiber especially at the cervical root region. CRL showed a stronger relationship than PL with MDL, as a variation in the values of the nerve conduction velocity and/or length of the arms had a greater influence on the PL than the CRL, than we anticipated.

It was not clear whether a disturbance in axonoplasmic flow following cervical root compression or retrograde degeneration related to the severity of CTS contributed to the conduction disturbance throughout the median nerve. We concluded that the retrograde degeneration had more influence because the CRL of the ulnar nerve, which derives from the same roots as the median nerve, was not prolonged. Chang et al. measured a forearm conduction velocity and a wrist-palm conduction in relatively severe CTS patients, mild CTS patients and healthy subjects, and found a direct-conduction velocity that reflects the velocity of two fibers passing through the carpal tunnel and another passing outside the carpal tunnel from an in an indirect -conduction velocity of only one fiber passing through the carpal tunnel. They concluded that retrograde axonal atrophy or retroconduction slowing could explain the motor conduction slowing of the median nerve in CTS patients. Their finding coincided well with our findings [29,30]. Besides these facts, we suspect two further reasons for the prolongation of CRL as follows, 1) there could be subclinical foramen stenosis with a spondylotic change, 2) the roots change direction at spur and subclinical kinking stress can exist with or without foramen stenosis, and only CRL of the more susceptible median nerve was prolonged in CTS patients.

If the term of the DCH indicates a condition in which one focal compression causes a conduction disturbance at another site more severely than expected from the linear relationship, #1 was not proven. If it means a condition in which a focal compression site influences other regions within a linear relationship, #1 was proven in the current study.

### Discussion about #2

Dual regions of compression on the nerve fiber exaggerate the total damage.

Phalen described that thenar atrophy often proceeds hypesthesia in the median distribution for many months or many years and the onset of thener muscle atrophy was always gradual. Also if the paralysis had existed for more than one year, the outlook for recovery after decompression of the median nerve was poor [31]. The amplitude of the compound muscle action potential reflects the structure of the motor unit: diameter, distribution and number of muscle fibers [32]. The CMAP amplitude is reduced in axon loss neuropathies and demyelinative neuropathies when a demyelinative lesion is interposed between the stimulus and recoding electrode [33]. Uchida demonstrated a strong relationship between the CMAP amplitude and the evoked mixed nerve action potential [18]. In this study, the CMAP amplitude approximated the severity of the CTS.

In 54 hands in the asymptomatic group, CRL measured in the median nerve was 1.10 ± 0.38 ms and that measured in the ulnar nerve was 1.19 ± 0.40 ms. Those values were within the normal range of several previous reports [34-37]. In this study, we temporally defined a prolonged CRL group that contained hands with a CRL longer than 1.48 ms

We divided the 143 symptomatic measurable hands into two groups according to the value of the CRL measured in the median nerve. One was a normal CRL group (n = 59 hands) and the other was a prolonged CRL group (n = 84 hands).

We compared the CMAP amplitude of the normal CRL group and that of the prolonged CRL group. No significant difference between CMAP amplitude of the normal CRL group and that of the prolonged CRL group was recognized (Fig 5.). MDL demonstrated a strong (r = 0.58, p < 0.0001, Fig. 3), while CRL showed a clear (r = 0.59, p < 0.0001, Fig. 4), relationship with the amplitude of CMAP. Therefore, it was suggested that dual lesion entrapment did not unexpectedly exaggerate the damage, but be rather indicated the severity of each lesion. #2 could not be proven in the current study. The degree of severity was within the expected range linear relationship among MDL, CRL and CMAP amplitude.

## Conclusion

If the term of the DCH represents an unexpectedly exaggerated degree, DCS in the CTS patients is rare. But, if the term DCH means only a linear relationship, DCH should not be dismissed. In either event, we must consider the damage at another site on the same nerve when diagnosig patients with palsy.

## Competing interests

The authors declare that they have no competing interests.

## Authors' contributions

RS, HK and KY performed the medical examinations and recorded the patients' symptoms. RS measured the nerve conduction and prepared the draft of the manuscript. HK and KY supervised his preparetion.
